# Burden of Liver Disease among Community-Based People Who Inject Drugs (PWID) in Chennai, India

**DOI:** 10.1371/journal.pone.0147879

**Published:** 2016-01-26

**Authors:** Sunil S. Solomon, Aylur K. Srikrishnan, Allison M. McFall, M. Suresh Kumar, Shanmugam Saravanan, Pachamuthu Balakrishnan, Suniti Solomon, David L. Thomas, Mark S. Sulkowski, Shruti H. Mehta

**Affiliations:** 1 Department of Medicine, Johns Hopkins School of Medicine, Baltimore, Maryland, United States of America; 2 Department of Epidemiology, Johns Hopkins Bloomberg School of Public Health, Baltimore, Maryland, United States of America; 3 YR Gaitonde Centre for AIDS Research and Education, Chennai, India; Drexel University College of Medicine, UNITED STATES

## Abstract

**Background and Objective:**

We characterize the burden of liver disease in a cohort of PWID in Chennai, India, with a high prevalence of HCV.

**Materials and Methods:**

1,042 PWID were sampled through community outreach in Chennai. Participants underwent fasting blood draw, questionnaire and an examination that included liver stiffness assessment using transient elastography (Fibroscan) and assessment of steatosis via ultrasound.

**Results:**

The median age was 39 years, all were male, 14.8% were HIV infected and 35.6% were HCV antibody positive, of whom 78.9% were chronically infected (HCV RNA positive). Median liver stiffness was 6.2 kPA; 72.9% had no evidence of or mild stiffness, 14.5% had moderate stiffness, and 12.6% had evidence of severe stiffness/cirrhosis. Prevalence of severe stiffness/cirrhosis was significantly higher among persons who were older, had a longer duration of injecting drugs, higher body mass index, higher prevalence of insulin resistance, higher prevalence of steatosis, higher HCV RNA levels and evidence of alcohol dependence. An estimated 42.1% of severe stiffness/cirrhosis in this sample was attributable to HCV. 529 (53.0%) had some evidence of steatosis. Prevalence of steatosis was higher among those who had larger waist circumference, insulin resistance, higher HDL cholesterol and a history of antiretroviral therapy.

**Conclusions:**

We observed a high burden of liver disease in this relatively young cohort that was primarily driven by chronic HCV infection, metabolic factors (insulin resistance and steatosis) and heavy alcohol use. Interventions to improve access to HCV treatment and reduce alcohol use are needed to prevent further progression of liver disease.

## Introduction

In high-income countries, mortality due to liver disease has eclipsed HIV-associated mortality [1]. As access to antiretroviral therapy (ART) for HIV continues to improve globally, a similar pattern will follow in low-and-middle-income settings (LMICs) where an estimated 90% of the 185 million HCV-infected persons reside [2]. The advent of directly acting antivirals (DAAs) for HCV treatment promises a cure for all. However, cost and access challenges will make it logistically and financially impossible for all to be treated, particularly in LMICs [3]. Thus, it is critical to identify who is at risk for adverse outcomes due to HCV such that they can be prioritized for treatment.

There are limited epidemiologic data on the burden of HCV and associated liver disease particularly in LMICs [4]. Moreover, these settings have a different background of comorbidities, co-infections and environmental characteristics, which may accelerate liver fibrosis progression and/or complicate treatment. The availability of non-invasive methods to stage liver disease allows for rapid estimation of liver disease in settings where liver biopsy is not feasible or acceptable. Transient elastrography has been demonstrated to be an accurate method for staging liver fibrosis for multiple underlying etiologies and predicting future adverse clinical events [5].

India has an estimated population HCV prevalence of 1–2% [6], an estimated 3 million opiate users [7] and an estimated 1.1 million PWID [8]. We characterize the burden of liver disease in a cohort of PWID in Chennai, India.

## Materials and Methods

### Study Population

The study was conducted through the YR Gaitonde Center for Substance Abuse Research (YRGCSAR), which was established in 2005 to conduct community-based research among PWID in Chennai. YRGCSAR is part of the YRG Medical Educational and Research Foundation, which includes the YR Gaitonde Centre for AIDS Research and Education (YRGCARE), which has been involved with HIV-related research since the mid-1990’s and has provided care to more than 20,000 individuals living with HIV/AIDs and associated co-infections. In a previous cohort of PWID in Chennai, we documented a high prevalence of HIV and HCV infection [9, 10]. The goal of this study was to further understand the burden and co-factors of liver disease in a population with high HIV and HCV burden by following a cohort of current and former PWID (The Chennai HIV, HCV and Eeral [liver disease] study [CHHEERS]). Accordingly, we recruited a convenience sample of PWID through community outreach in Chennai, India. This process included a series of community meetings with current and former PWID and representatives from organizations serving PWID locally. Outreach workers subsequently visited areas where PWID congregate and accompanied potential participants to the study clinic. Participants could also self-refer if they either saw a flyer for the study or heard about the study from a field worker or another participant. Participants had to be ≥18 years old, provide informed consent, and report a history of drug injection in the prior five years. Because our intention was to follow the cohort over time, we also required that participants have no intention of migrating for two years. Overall, 1,324 participants were screened of whom 1,062 were eligible and 1,042 enrolled. Nearly all screened participants found ineligible (96%) were excluded due to drug use criteria.

### Study Procedures

All participants came to the clinic after an overnight fast. Participants provided written informed consent, underwent a blood draw and responded to a questionnaire that collected information on socio-demographics, past and current substance use and HIV, HCV and hepatitis B testing and treatment history. A nurse collected information on medications, clinical history and vital signs and a physician collected information on signs of liver disease, anthropometrics and performed a liver stiffness assessment via transient elastography. All underwent ultrasound of the liver to characterize steatosis.

### Transient Elastography

Transient elastography was performed using a FibroScan machine (EchoSens, Paris, France) [11]. Briefly, this technology involves pulse-echo ultrasonography acquisitions, which measure the velocity of a shear wave propagated through the liver. Results are instantaneously received as a single, quantitative variable of liver stiffness measurement (LSM), reported in kilopascals (kPa). Two certified operators trained by the manufacturer performed all assessments with a single device. Only examinations with ≥10 discrete valid measurements, a success rate ≥60% and limited variability (interquartile range (IQR) of measures divided by the median value ≤30%) were considered valid [12]. Of the 1,042 enrolled, 1,029 participants underwent elastography. Thirteen did not undergo elastography because of ascites (n = 4), other medical conditions (n = 4), or unavailability of fibroscan machine (n = 5). An additional 12 had invalid results leaving 1,017 for analysis.

### Ultrasound

Ultrasound was performed by a radiologist using a high-resolution B-mode ultrasonography system (Logic 400, GE, Milwaukee, WI) with an electric linear transducer midfrequency of 3–5 MHz [13]. Fatty liver was defined as the presence of a pattern consistent with ‘bright liver,’ with evidence of increased ultrasonographic contrast between the hepatic and renal parenchyma, vessel blurring, and narrowing of the lumen of the hepatic veins. If present, fatty liver severity was based on standard criteria: 1) Grade 1 (mild): slightly increased liver echogenicity with normal vessels and absent posterior attenuation; 2) Grade 2 (moderate): moderately increased liver echogenicity with partial dimming of vessels and earlier posterior attenuation; and Grade 3 (severe): diffusely increased liver echogenicity with absence of visible vessels and heavy posterior attenuation [14].

### Laboratory testing

HIV serostatus was determined using double ELISA testing (Murex HIV-1.2.O, Abbott Murex, UK and Vironostika^®^ HIV Uni-form II Ag/Ab, Biomérieux, The Netherlands). CD4+ count was estimated using the FlowCARE™ PLG CD4 (CD45-FITC/CD4-PE) assay (Beckman Coulter, CA, USA) and HIV-1-RNA with RealTime HIV-1 assay (Abbott Laboratories, Abbott Park, Illinois, USA). HCV antibody testing was performed using the Genedia HCV ELISA 3.0 (Green Cross Medical Science, Chungbuk, Korea). HCV RNA was ascertained using the RealTime HCV assay (Abbott Molecular Inc., Des Plaines, IL, USA). Chronic HBV infection was measured by presence of hepatitis B surface antigen (Hepanostika HBsAg Uniform II, Biomérieux, The Netherlands). Plasma glucose, tryglicerides, total cholesterol, HDL cholesterol, LDL cholesterol were measured using an enzymatic methods (Olympus AU400; Olympus Diagnostica, Tokyo, Japan). Plasma insulin was measured by immunoassay (ELISA) using a Bioscience kit (Monobind kit, Monobind Inc., Lake Forest, CA, USA). Homeostasis model assessment (HOMA–IR) was calculated as (fasting plasma glucose (mmol/L) x fasting insulin (μU/mL)/22.5).

### Statistical Analysis

We present a cross-sectional analysis of data from the baseline visit. Analyses were restricted to participants with complete data at this visit on covariates of interest: valid fibroscan score, ultrasound, HCV and HIV status. Demographic, behavioral clinical and laboratory characteristics of persons were compared by HCV RNA status and liver stiffness using Fisher’s exact tests for categorical variables and Kruskal-Wallis tests for continuous variables. Persons were classified as either having no/mild stiffness (LSM < 8.5 kPA), moderate stiffness (8.5–12.3 kPA) or severe stiffness/cirrhosis (≥12.3 kPA) based on cutoffs established in India and the US [15–17]. Correlates of moderate stiffness and severe stiffness/cirrhosis were examined using multinomial logistic regression (reference group = no/mild stiffness). Factors were considered for multivariable models based on prior evidence and statistical associations in univariable models (p<0.10). Variables were retained in final models if associated with either outcome at p<0.05. Sensitivity analyses were conducted to explore the impact of different published cutoffs from India and other settings (7–9kPa for moderate stiffness and 12–15kPa for severe stiffness/cirrhosis). Using the final multivariable models, we calculated the population attributable fractions of severe stiffness/cirrhosis for the total population and those chronically infected with HCV [18]. Persons with any steatosis (Grade 1, 2 or 3) were compared to those with no steatosis (Grade 0) using logistic regression. Analyses were conducted in STATA Version. 13.0 (College Station, Texas).

### Ethical Clearances

This study was approved by the Johns Hopkins and YRGCARE Institutional Review Boards.

## Results

The median age of the 998 participants with complete data was 39 (interquartile range (IQR), 32–45). All were male and 57.5% had primary school education only or less. Though 94.7% reported a history of injecting heroin, only 13.4% reported injection in the prior 6 months. 90.5% reported a lifetime history of marijuana use and 94.3% ever smoking cigarettes. At baseline, 38.0% reported drinking 7 or more drinks per day and 57.6% had evidence of alcohol dependence on AUDIT. 148 (14.8%) were HIV-infected and 355 (35.6%) were HCV antibody positive.

Overall, 280 (78.9%) of the 355 HCV antibody persons were chronically infected. Compared to the other two groups, those with chronic HCV infection were significantly older and significantly more likely to have injected buprenorphine and other prescription drugs, have used non-injection drugs and to have smoked cigarettes. Those chronically infected with HCV were significantly more likely to be HIV positive and have a history of tuberculosis and significantly less likely to be chronically infected with hepatitis B and have alcohol dependence (Table 1, p<0.05 for all).

**Table 1:** Characteristics of study population by HCV status at baseline (N = 998).

	HCV-uninfected	HCV-infected		
	(N = 643)	Viral clearance (N = 75)	Persistent (N = 280)	p-value^1^
**Median age**	39 (32–45)	36 (29–44)	41 (33–45)	<0.001
**Male**	643 (100)	75 (100)	280 (100)	—
**Education**				
*None or primary*	389 (60.5)	39 (52.0)	146 (52.1)	<0.01
*Secondary*	9 (15.2)	10 (13.3)	31 (11.1)	
*High School*	98 (15.2)	18 (24.0)	62 (22.1)	
*Vocational/University/Graduate*	58 (9.0)	8 (10.7)	41 (14.6)	
**Median monthly income, US dollars**	90(67–135)	75(45–105)	90(67–142)	0.03
**Lifetime drug injection**				
*Heroin*	603 (93.8)	73 (97.3)	269 (96.1)	0.25
*Cocaine/methamphetamine*	13 (2.0)	8 (10.7)	17 (6.1)	<0.001
*Buprenorphine*	323 (50.2)	64 (85.3)	258 (92.1)	<0.001
*Prescription opiates other*	80 (12.4)	31 (41.3)	111 (39.6)	<0.001
*Sedatives*	218 (33.9)	50 (66.7)	200 (71.4)	<0.001
**Lifetime non-injection drug use**				
*Marijuana*	562 (87.4)	69 (92.0)	272 (97.1)	<0.001
*Heroin*	302 (47.0)	56 (74.7)	238 (85.0)	<0.001
*Cocaine/methamphetamine*	18 (2.8)	4 (5.3)	9 (3.2)	0.44
*Buprenorphine*	81 (12.6)	28 (37.3)	103 (36.8)	<0.001
*Prescription opiates other*	54 (8.4)	16 (21.3)	46 (16.4)	<0.001
*Sedatives*	312 (48.5)	45 (60.0)	184 (65.7)	<0.001
**Median cigarettes smoked daily**	10 (5–15)	7 (4–10)	10 (5–20)	<0.001
**Drinks per day**				
*None*	62 (9.6)	17 (22.7)	66 (23.6)	<0.001
*1–4*	161 (25.0)	15 (20.0)	81 (28.9)	
*5–6*	160 (24.9)	13 (17.3)	44 (15.7)	
*7 or more*	260 (40.4)	30 (40.0)	89 (31.8)	
**AUDIT category**				
*No/mild alcohol use*	147 (22.9)	28 (37.3)	90 (32.1)	0.001
*Harmful/hazardous alcohol use*	116 (18.0)	5 (6.7)	37 (13.2)	
*Alcohol dependence*	380 (59.1)	42 (56.0)	153 (54.6)	
**Injection drug use in prior 6 months**	68 (10.6)	18 (24.0)	48 (17.1)	0.001
**HIV and treatment status**				
*Negative*	595 (93.0)	57 (76.0)	195 (69.6)	<0.001
*Positive and no ART*	14 (2.2)	9 (12.0)	56 (20.0)	
*Positive on ART*	31 (4.8)	9 (12.0)	29 (10.4)	
**Ever HCV treatment**†	NA	6 (8.0)	5 (1.8)	0.01
**Alternative medications for HIV**†	2 (4.4)	0 (0)	1 (1.2)	0.51
Alternative medications for HCV*	NA	1 (1.3)	6 (2.1)	1.000
**HBsAg positive**	45 (7.0)	13 (17.3)	13 (4.6)	<0.01
**History of tuberculosis**	84 (13.1)	18 (24.0)	72 (25.7)	<0.001
**History of dengue**	5 (0.8)	0 (0)	0 (0)	0.43
**History of chikungunya**	51 (7.9)	4 (5.3)	24 (8.6)	0.81
**History of malaria**	76 (11.8)	13 (17.3)	31 (11.1)	0.25

Data are presented as n (%) or median (interquartile range)

^1^Fisher’s exact test for categorical variables and Kruskal-Wallis for continuous variables

†Among HIV positives

*Among HCV antibody positives

AUDIT: Alcohol Use Disorders Identification Test; ART: antiretroviral therapy; HBsAg: surface antigen of the hepatitis B virus

Overall, 196 (19.6%) reported that a physician told them that they had liver disease including 55 (19.6%) of those chronically HCV-infected. In addition 141 (14.1%) had signs/symptoms of liver disease on exam (e.g., ascites) including 37 (13.2%) of those with chronic HCV. Signs/symptoms of liver disease were also significantly more common among those with severe stiffness/cirrhosis (27%, Table 2). Increasing liver stiffness was also significantly associated with increasing ALT, AST, GGT, total bilirubin, triglycerides, glucose, insulin, insulin resistance and with decreasing albumin, HDL and LDL cholesterol (p<0.05 for all comparisons).

**Table 2:** Clinical and laboratory parameters by stiffness stage (N = 998).

	No/mild Stiffness (N = 727)	Moderate Stiffness (N = 145)	Severe Stiffness/ Cirrhosis (N = 126)	p-value^1^
**Median age**	37 (31–44)	40 (33–45)	44 (38–47)	<0.001
**Injection drug use in prior 6 months**	104 (14.3)	17 (11.7)	13 (10.3)	0.43
**Alcohol use (drinks per day)**				
*None*	111 (15.3)	19 (13.1)	15 (11.9)	0.02
*1–4*	186 (25.6)	36 (24.8)	35 (27.8)	
*5–6*	176 (24.2)	22 (15.8)	19 (15.1)	
*7 or more*	254 (34.9)	68 (46.9)	57 (45.2)	
**AUDIT category**				
*No/mild alcohol use*	205 (28.2)	34 (23.5)	26 (20.6)	0.23
*Harmful/hazardous alcohol use*	119 (16.4)	21 (14.5)	18 (14.3)	
*Alcohol dependence*	403 (55.4)	90 (62.1)	82 (65.1)	
**HIV and treatment status**				
*Negative*	622 (85.9)	122 (84.1)	103 (81.8)	0.26
*Positive and no ART*	50 (6.9)	16 (11.0)	13 (10.3)	
*Positive on ART*	52 (7.2)	7 (4.8)	10 (7.9)	
**HBsAg positive**	44 (6.1)	12 (8.3)	15 (11.9)	0.06
**History of liver disease**	149 (20.5)	24 (16.6)	23 (18.3)	0.53
**Signs or symptoms of liver disease**	90 (12.4)	17 (11.7)	34 (27.0)	<0.001
**Any fat maldistribution**	110 (15.1)	17 (11.7)	25 (19.8)	0.18
**Moderate/severe fat maldistribution**	40 (5.5)	8 (5.5)	10 (7.9)	0.52
**Fat accumulation**	5 (0.7)	0 (0)	0 (0)	0.80
**Median body mass index, kg/m**^**2**^	19.3 (17.7–22.4)	20.3 (18.1–23.7)	20.7 (18.5–24.4)	<0.001
**Median waist circumference, cm**	75.0 (70.0–85.0)	79.0 (73.8–90.0)	82.2 (75.0–92.0)	<0.001
**Median hip circumference, cm**	78.0 (73.2–86.0)	80.0 (74.5–88.0)	82.2 (75.0–89.8)	<0.001
**Median mid-arm circumference, cm**	25.4 (23.4–28.0)	26.0 (23.7–28.5)	26.2 (23.4–28.4)	0.17
**Median CD4 cell count, cells/mm**^**3**^†	359 (239–491)	426 (272–536)	254 (169–435)	0.12
**Median log**_**10**_ **HIV RNA, copies/ml**†	3.5 (1.6–4.6)	4.3 (3.1–5.5)	4.2 (2.9–5.1)	0.15
**Median log**_**10**_ HCV RNA, copies/ml*	6.4 (5.8–6.8)	6.3 (5.9–6.7)	6.4 (5.9–6.6)	0.65
**Median ALT, mg/dl**	23 (15–39)	43 (24–82)	61 (30–103)	<0.001
**Median AST, mg/dl**	29 (23–47)	59 (31–108)	82 (55–156)	<0.001
**Median GGT, mg/dl**	32 (21–64)	70 (34–203)	173 (76–313)	<0.001
**Median albumin g/dl**	4.2 (4.0–4.5)	4.2 (3.9–4.4)	4.0 (3.5–4.3)	<0.001
**Median bilirubin, mg/dl**	0.7 (0.5–1.0)	0.8 (0.6–1.1)	0.9 (0.7–1.3)	<0.001
**Median total protein, g/dl**	7.5 (7.1–8.0)	7.6 (7.1–8.0)	7.8 (7.3–8.2)	<0.01
**Median total cholesterol, mg/dl**	170 (141–199)	169 (140–203)	153 (124–188)	<0.01
**Median HDL, mg/dl**	42 (34–54)	43 (33–60)	37 (28–47)	<0.001
**Median LDL, mg/dl**	101 (79–124)	99 (76–127)	89 (70–121)	0.04
**Median triglycerides, mg/dl**	89 (64–126)	95 (71–140)	101 (78–143)	<0.01
**Median insulin, μU/ml**	5 (3–10)	7 (3–12)	8 (4–17)	<0.001
**Median glucose, mg/dl**	81 (74–90)	86 (78–97)	89 (81–105)	<0.001
**Median HOMA IR**	1.1 (0.5–2.0)	1.4 (0.7–2.7)	1.9 (0.8–4.2)	<0.001
**Steatosis**				
*None*	371 (51.0)	52 (35.9)	46 (36.5)	<0.001
*Mild*	278 (38.2)	67 (46.2)	58 (46.0)	
*Moderate*	78 (10.7)	26 (17.9)	22 (17.5)	

Data are presented as n (%) or median (interquartile range)

^1^Fisher’s exact test for categorical variables and Kruskal-Wallis for continuous variables

†Among HIV positives

*Among HCV antibody positives

ALT: alanine aminotransferase; AST: aspartate aminotransferase; GGT: Gamma-glutamyltransferase; HDL: high-density lipoprotein; LDL: low-density lipoprotein; HOMA IR: homeostasis model assessment-estimated insulin resistance score

### Liver stiffness

**Fig 1:**
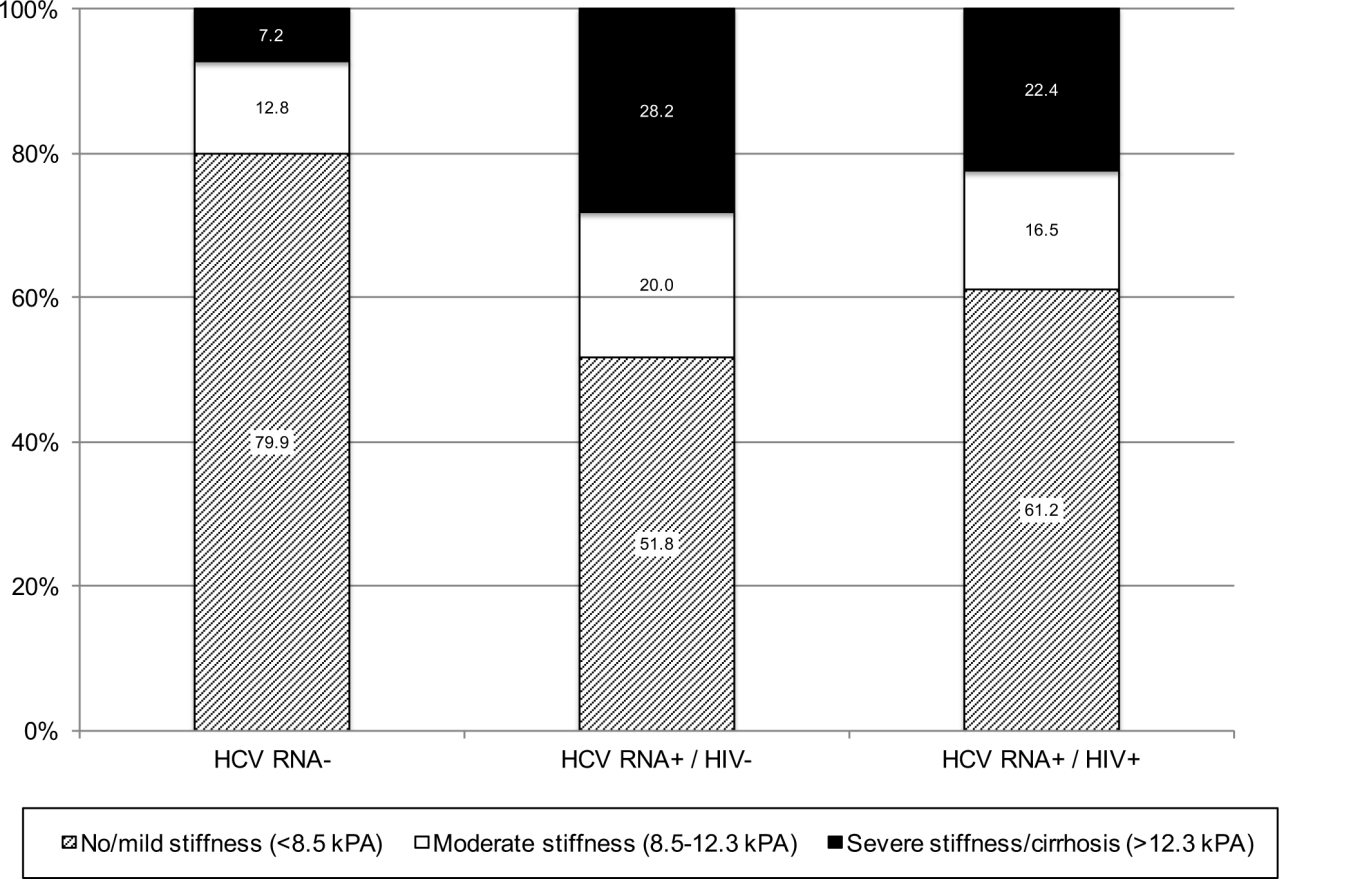
Distribution of liver stiffness by HIV and HCV status.

The median LSM was 6.2 kPA (IQR, 4.9, 8.8); 727 (72.9%) had no/mild stiffness, 145 (14.5%) had moderate stiffness and 126 (12.6%) had severe stiffness/cirrhosis. The prevalence of moderate and severe stiffness were significantly higher among persons who were chronically HCV infected compared to the other two groups (Fig 1). In univariable analysis, compared to those with no stiffness, those with moderate stiffness were also significantly older, had injected drugs for longer duration, higher BMI, higher prevalence of insulin resistance, higher prevalence of steatosis and were more likely to have HCV RNA >5 log10 IU/ml (Table 3). In multivariable analysis, all of these factors remained significantly associated with moderate stiffness. In univariable analysis, compared to those with no stiffness, those with severe stiffness/cirrhosis were significantly older, had a longer duration of injecting drugs, higher BMI, higher prevalence of insulin resistance, higher prevalence of steatosis, higher HCV RNA levels, and a higher prevalence of chronic HBV. In multivariable analysis, older age (OR: 1.39; 95% CI: 1.16, 1.65), higher BMI (OR: 1.17; 95% CI: 1.07, 1.29), insulin resistance (OR: 1.98; 95% CI: 1.25, 3.13), steatosis (OR for moderate compared to no steatosis: 2.82; 95% CI: 1.55, 4.64), higher HCV RNA (OR for HCV RNA >6 log_10_IU/ml vs. HCV un-infected: 6.77; 95% CI: 4.04, 11.3), chronic HBV (OR: 2.57; 95% CI: 1.26, 5.27) and alcohol dependence (OR: 2.68; 95% CI: 1.55, 4.64) were significantly associated with severe fibrosis/cirrhosis. Neither HIV status nor CD4 cell count, HIV RNA level or antiretroviral therapy use were associated with moderate or severe stiffness/cirrhosis.

**Table 3:** Correlates of liver stiffness and cirrhosis at baseline.

	Ratio of Unadjusted Odds Ratios† (95% CI)		Ratio of Adjusted Odds Ratios† (95% CI)	
	Moderate stiffness	Severe Stiffness/ Cirrhosis	Moderate stiffness	Severe Stiffness/ Cirrhosis
**Age (per 5 years)**	1.12 (1.01–1.24)	1.43 (1.28–1.60)	0.95 (0.81–1.11)	1.39 (1.16–1.65)
**Age at initiation of drug injection (per 5 years)**	0.93 (0.81–1.08)	1.20 (1.05–1.37)	—	—
**Years of injection drug use (per 5 years)**	1.21 (1.08–1.35)	1.35 (1.20–1.52)	1.18 (1.00–1.40)	0.98 (0.81–1.17)
**Injection drug use in prior 6 months**	0.80 (0.46–1.37)	0.69 (0.37–1.27)	—	—
**Marijuana use in prior 6 months**	0.70 (0.49–1.00)	0.61 (0.42–0.89)	—	—
**AUDIT category**				
*No/mild alcohol use*	REF	REF	REF	REF
*Harmful/hazardous alcohol use*	1.06 (0.59–1.92)	1.19 (0.63–2.27)	1.45 (0.77–2.72)	2.30 (1.10–4.80)
*Alcohol dependence*	1.35 (0.88–2.07)	1.60 (1.00–2.57)	1.67 (1.05–2.65)	2.68 (1.55–4.64)
**BMI (per 2 kg/m**^**2**^**)**	1.13 (1.04–1.23)	1.20 (1.10–1.30)	1.10 (1.01–1.21)	1.17 (1.07–1.29)
**Insulin resistance (HOMA IR>2)**	1.88 (1.29–2.74)	2.63 (1.78–3.89)	1.69 (1.12–2.55)	1.98 (1.25–3.13)
**HDL (per 20 mg/dl)**	1.12 (0.94–1.33)	0.52 (0.39–0.69)	—	—
**LDL (per 20 mg/dl)**	1.00 (0.90–1.11)	0.86 (0.76–0.96)	—	—
**Total cholesterol (per 20 mg/dl)**	1.03 (0.96–1.11)	0.85 (0.78–0.93)	—	—
**Steatosis**^				
*Normal*	REF	REF	REF	REF
*Mild*	1.72 (1.16–2.55)	1.68 (1.11–2.55)	1.58 (1.05–2.39)	1.42 (0.89–2.26)
*Moderate*	2.38 (1.40–4.04)	2.27 (1.29–4.00)	2.44 (1.40–4.26)	2.82 (1.55–4.64)
**HIV status**				
*Negative*	REF	REF	—	—
*Positive*, *no ART*	1.63 (0.90–2.96)	1.57 (0.82–2.99)		
*Positive*, *ART use*	0.69 (0.30–1.55)	1.16 (0.57–2.36)		
**HCV positive**				
*HCV uninfected*	REF	REF	REF	REF
*HCV RNA<2*.*8 log10 IU/ml*	0.57 (0.24–1.37)	2.50 (1.24–5.01)	0.48 (0.20–1.17)	1.95 (0.90–4.26)
*HCV RNA 2*.*8–5 log10 IU/ml*	0.42 (0.06–3.26)	3.69 (1.16–11.7)	0.34 (0.04–2.74)	3.86 (1.06–14.1)
*HCV RNA 5–6 log10 IU/ml*	3.08 (1.52–6.26)	9.80 (4.98–19.3)	2.75 (1.31–5.74)	9.70 (4.65–20.2)
*HCV RNA > 6 log10 IU/ml*	2.05 (1.33–3.16)	5.98 (3.77–9.50)	2.10 (1.32–3.35)	6.77 (4.04–11.3)
**HBsAg positive**	1.40 (0.72–2.72)	2.10 (1.13–3.90)	1.59 (0.80–3.19)	2.57 (1.26–5.27)
**History of tuberculosis**	0.79 (0.48–1.29)	1.09 (0.67–1.77)	—	—
**History of dengue or chikungunya**	0.91 (0.47–1.78)	1.06 (0.54–2.08)	—	—
**History of malaria**	0.98 (0.56–1.70)	1.07 (0.60–1.89)	—	—

†Base outcome is no/mild stiffness

^Assessed using ultrasound

AUDIT: Alcohol Use Disorders Identification Test; BMI: body mas index; HOMA IR: homeostasis model assessment-estimated insulin resistance score; HDL: high-density lipoprotein; LDL: low-density lipoprotein; HBsAg: surface antigen of the hepatitis B virus

**Fig 2:**
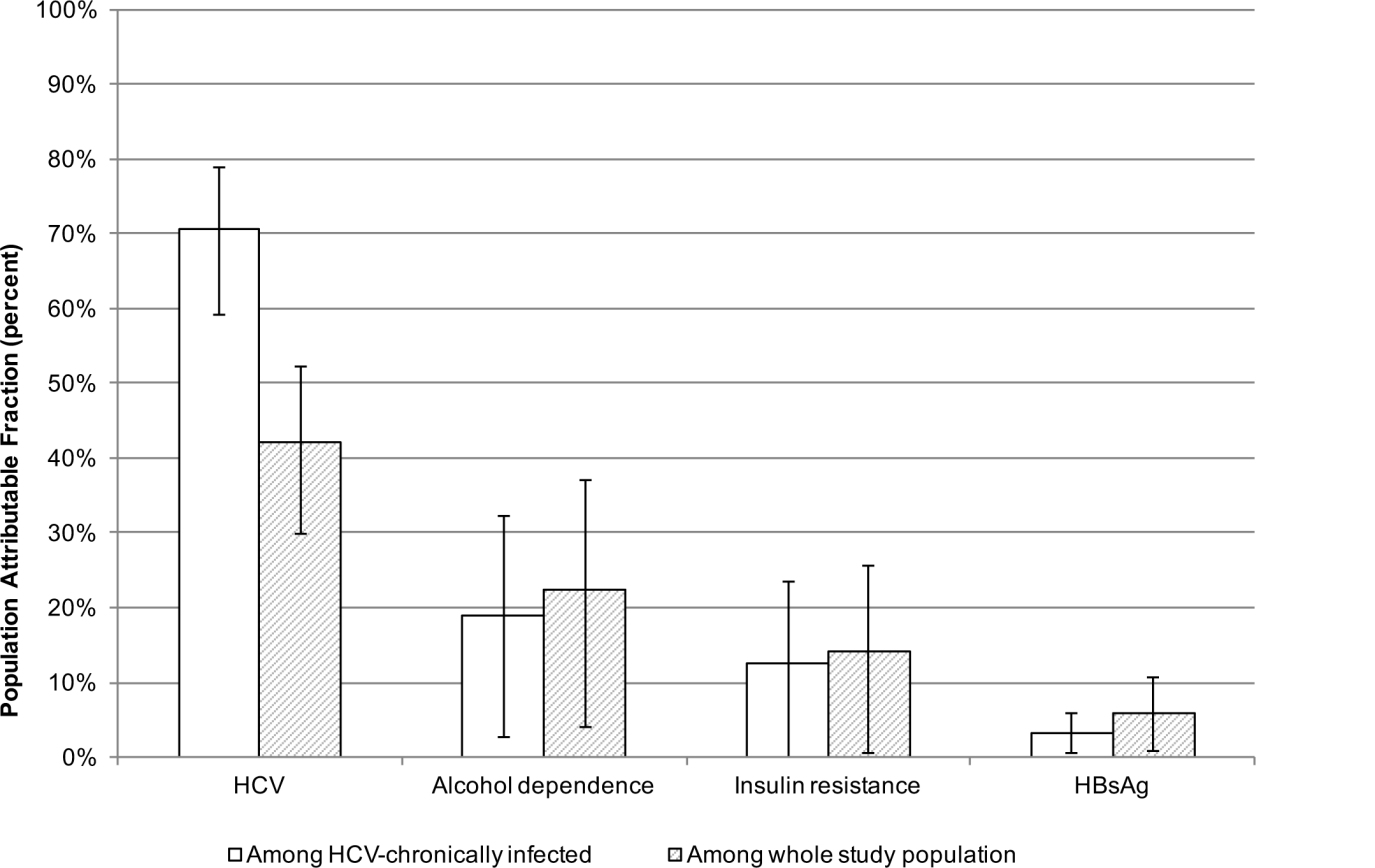
Population attributable fractions of severe liver stiffness/cirrhosis among total population and persons with chronic HCV infection.

Among persons chronically infected with HCV, 70.6% of the cirrhosis was attributable to chronic HCV (Fig 2). Among the total sample, 42.1% of the cirrhosis was attributable to chronic HCV. Alcohol use was responsible for 18.9% of the cirrhosis among those chronically infected with HCV and 22.4% of that in the total sample.

### Liver steatosis

469 (47.0%) had no steatosis, 403 (40.4%) had mild steatosis, 126 (12.6%) moderate steatosis and none had evidence of severe steatosis. Steatosis prevalence did not differ by whether persons were chronically infected with HCV (54.2% vs. 50.0%, p = 0.26). Compared to those with no steatosis, those with any steatosis were significantly older, had longer duration of injection drug use, higher BMI, waist circumference, hip circumference, insulin resistance, and higher HDL and LDL cholesterol (Table 4). A history of tuberculosis was associated with lower odds of steatosis (OR: 0.65; 95% CI: 0.47, 0.91). In multivariable analysis, larger waist circumference (OR: 1.17; 95% CI: 1.10, 1.25), insulin resistance (OR: 1.49; 95% CI: 1.09, 2.02), higher HDL cholesterol (OR: 1.63; 95% CI: 1.39, 1.91) and antiretroviral therapy use (OR: 1.82; 95% CI: 1.04, 3.19) were independently associated with liver steatosis. Tuberculosis remained negatively associated with steatosis (OR: 0.67, 95% 0.46, 0.98).

**Table 4:** Correlates of liver steatosis* at baseline.

	Unadjusted Odds Ratio (95% CI)	Adjusted Odds Ratio (95% CI)
**Age (scaled by 5 years)**	1.07 (1.00–1.15)	1.05 (0.98–1.13)
**Age at initiation of drug injection (per 5 years)**	1.01 (0.92–1.11)	—
**Years of injection drug use (per 5 years)**	1.09 (1.01–1.18)	—
**Injection drug use in prior 6 months**	0.84 (0.58–1.21)	—
**Marijuana use in prior 6 months**	0.65 (0.51–0.84)	—
**AUDIT category**		
*No/mild alcohol use*	REF	—
*Harmful/hazardous alcohol use*	0.62 (0.41–0.92)	
*Alcohol dependence*	1.19 (0.89–1.59)	
**BMI (per 2 kg/m**^**2**^**)**	1.14 (1.07–1.22)	—
**Waist circumference (per 5 cm)**	1.16 (1.09–1.23)	1.17 (1.10–1.25)
**Hip circumference (per 5 cm)**	1.22 (1.14–1.31)	—
**Insulin resistance (HOMA IR>2)**	1.72 (1.30–2.28)	1.49 (1.09–2.02)
**HDL cholesterol (per 20 mg/dl)**	1.41 (1.22–1.63)	1.63 (1.39–1.91)
**LDL cholesterol (per 20 mg/dl)**	1.11 (1.03–1.20)	—
**Triglycerides (per 20 mg/dl)**	1.02 (1.00–1.05)	—
**History and/or current high blood pressure**†	1.36 (0.61–3.07)	—
**HIV and treatment status**		
*Negative*	REF	REF
*Positive*, *no antiretroviral therapy*	0.77 (0.48–1.22)	1.36 (0.83–2.25)
*Positive*, *antiretroviral therapy use*	1.07 (0.65–1.75)	1.82 (1.04–3.19)
**HCV Positive**		
*HCV uninfected*	REF	—
*HCV RNA<2*.*8 log10 IU/ml*	0.89 (0.56–1.41)	
*HCV RNA 2*.*8–5 log10 IU/ml*	0.94 (0.38–2.33)	
*HCV RNA 5–6 log10 IU/ml*	1.25 (0.72–2.16)	
*HCV RNA > 6 log10 IU/ml*	0.77 (0.56–1.06)	
**HBsAg positive**	1.39 (0.85–2.28)	—
**History of tuberculosis**	0.65 (0.47–0.91)	0.67 (0.46–0.98)

*Steatosis: Grade 1, 2 or 3 found on ultrasound

†Ever told by doctor that have high blood pressure or ever taken medications for high blood pressure

AUDIT: Alcohol Use Disorders Identification Test; BMI: body mas index; HOMA IR: homeostasis model assessment-estimated insulin resistance score; HDL: high-density lipoprotein; LDL: low-density lipoprotein; HBsAg: surface antigen of the hepatitis B virus

## Discussions and Conclusions

In this relatively young population of PWID with a high prevalence of chronic HCV, we identified a high burden of liver disease. Among persons with chronic HCV, half had evidence of at least moderate stiffness and nearly 30% had evidence of severe stiffness or cirrhosis. Beyond HCV, metabolic factors and alcohol use were strong cofactors of stiffness. As this population continues to age, the burden of liver disease will continue to increase without substantial improvements in access to antiviral therapy for HCV.

Our data represents one of the first characterizations of liver disease among PWID in a LMIC setting. Compared to prior reports among PWID, the prevalence of liver stiffness in our sample appears higher. For example, in a US-based study of PWID in Baltimore, 13.9% of those chronically infected with HCV had cirrhosis and 10.6% had clinically significant fibrosis [15, 16] but not cirrhosis. Levels among persons chronically infected with HCV in our sample were more than double despite the median age being seven years younger than the US-based cohort. Our results are comparable to other samples in India among the general population and liver clinic patients. Prior studies in India have suggested higher than expected liver stiffness levels in patients without evidence of histologic fibrosis or inflammation, suggesting that liver stiffness increases in Indian patients prior to the onset of fibrosis [17, 19]. While we did not have histologic data to confirm these findings, the higher than expected values of liver stiffness support this conjecture.

The high prevalence of stiffness may also reflect a predominance of metabolic abnormalities, which could be important drivers of stiffness in this population. Overall, 30% had evidence of insulin resistance and 53% had evidence of mild/moderate steatosis–this is despite a median BMI of only 20 compared to 25–26 in comparable studies in Western setting [20, 21]. The reasons for this metabolic profile are multifactorial. There are some histologic data from the general Indian population on non-alcoholic fatty liver disease (NAFLD), which suggest that prevalence appears comparable to the West despite lower prevalence of obesity [22]. It is also well recognized that Asian Indians have a high prevalence of insulin resistance and related metabolic disturbances (central adiposity, raised triglycerides, low HDL cholesterol and type 2 diabetes) [23]. While the reasons for increased insulin resistance among Indians are not completely understood, family studies and a genome-wide association study (GWAS) of insulin resistance suggest a strong genetic component [24], with insulin resistance among Asian men occurring at lower BMI compared to other groups [25]. Regardless of etiology, the high prevalence of these factors in persons with and at risk for chronic HCV infection, suggests that liver disease may also progress more rapidly in this group.

Aside from HCV and metabolic factors, one of the strongest correlates of stiffness in this cohort was alcohol use. We observed an alarmingly heavy burden of alcohol use in this cohort with 58% having evidence of alcohol dependence by AUDIT and 38% reporting more than 7 drinks per day. We have previously demonstrated that in this population, injection drug use has declined over time with reciprocal increases in alcohol use [9]. This is despite repeated counseling on the harms associated with alcohol use particularly for persons chronically infected with hepatitis C. In the US, the Centers for Disease Control now recommend that a diagnosis of chronic hepatitis C be accompanied by a brief alcohol intervention [26]. Such recommendations should also be considered for a setting like India with the recognition that more intensive intervention will be required in some cases.

Interestingly, while HIV has consistently been associated with more rapid liver disease progression among HCV-infected persons, we failed to observe any association between HIV and liver stiffness even among the most immunocompromised (CD4<200 cells/μl). There are multiple possible explanations for this. First, India has predominantly HIV subtype C and HCV genotype (GT) 3 whereas most prior characterizations have been performed in settings with HIV subtype B and HCV GT1. Second, in prior studies in this same population, we observed extremely high mortality rates particularly among HIV positive PWID with low CD4 cell counts (34.5 per 100 person-years) [27, 28]. Thus, it is possible that the lack of observation of an association reflects competing mortality due to AIDS. In fact, this replicates what was observed in studies in the US where in the early years of HAART availability, we failed to detect an association between HIV and liver disease [29] but did more than a decade later when HAART access had dramatically improved [30].

We observed a statistically significant association between chronic hepatitis B virus infection and cirrhosis that was independent of other factors. Overall, 7% of this population was chronically infected with hepatitis B. Interestingly, the prevalence of HBsAg was four-fold higher in persons who had cleared HCV as compared to those with persistent infection and appeared to explain the higher prevalence of cirrhosis among those who had cleared HCV. Moreover, it appears that HCV clearance rate was 50% in HBsAg positive participants compared to 81% in HBsAg negative participants. In general, coinfection with hepatitis B and C has been associated with more severe liver disease [31, 32], but there has also been evidence of reciprocal inhibition of the viral genomes, with each preventing or decreasing the ability of the other to replicate [33]. However, which virus dominates tends to be different in different settings and potentially determined by which is acquired first. While the most common pattern observed has been a dominance of HCV with higher levels of HCV RNA combined with low or undetectable levels of HBV DNA and negative hepatitis B e antigen [34], others particularly those among Asian settings and patients have observed HBV dominance [35–38]. The majority of these studies have focused on measuring HCV RNA and HBV DNA levels among persons chronically infected with both viruses. Less is known about the impact of HBsAg positivity and HCV clearance and vice versa. A single study demonstrated that superinfection with hepatitis B virus in 24 chronic HCV carriers led to spontaneous clearance of HCV in 6 persons [39]. Unfortunately, we did not have data on other markers of HBV, including HBV core and surface antibodies, HBV DNA levels and HBV e antigen (HBeAg) to further elucidate the mechanism of action but further study is warranted.

In India, access to curative therapies is challenging. Sofosbuvir and more recently a fixed-dose combination of sofosbuivir and ledipasvir have recently been licensed in India and while at a reduced cost relative to resource-rich settings, it still remains out of reach for most PWID. Efforts need to be made to improve access to treatment particularly for those with evidence of severe fibrosis. However, it will be critical that these efforts include strategies to reduce alcohol use in this population in order to prevent further progression of liver disease.

We were limited by the cross-sectional nature of our data and by not having any data on liver biopsies to validate Fibroscan or ultrasound. We are not aware of a reason liver stiffness measures would correlate differently with fibrosis in India compare to the West. In the US and Western Europe, a systematic review identified the optimal cutoffs for mild/moderate fibrosis to be between 7.1 and 8.8 and for cirrhosis between 12.5 and 14.8 [40]. Fibroscan is being used elsewhere in India and there have been reports comparing Fibroscan to biopsy. We used the lower cutoff identified in a report by Das et al where the optimal cutoff for significant fibrosis was 8.5 kPa [17]. In this report no upper cutoff was identified for severe fibrosis/cirrhosis so we used the cutoff identified in our US-based studies which is consistent with that identified in the meta-analysis [29]. However, because higher cutoffs were identified by another report by Sharma and colleagues among 185 patients with multiple etiologies (10.0 kPA for mild/moderate fibrosis and 14.7 kPA for cirrhosis) [41], we conducted sensitivity analyses for different potential cutoffs of significant fibrosis and the range of prevalence was 7.8–30.3%. However, while the actual prevalence of fibrosis might be lower in this population, associations did not differ regardless of the cutoff used. Prior studies comparing ultrasound results to biopsy have found that ultrasound underestimates fatty liver disease when the total area of steatosis was <20% so if anything it is possible that the prevalence of steatosis in this population was underestimated. Additional limitations include the generalizability of our findings, which may be limited due to the convenience sampling, as well as the lack of women in our sample. Our team as well as others have previously demonstrated that there are few female PWID outside of Northeastern India [42–44]; and in other settings some sex differences with respect to fibrosis progression have been observed [45–47]. Moreover, PWID may be different from the general population with respect to other behaviors that influence liver disease.

In conclusion, we observed a significant burden of liver stiffness in this cohort of PWID with high prevalence of HCV infection. While the primary driver of fibrosis in this population was HCV, metabolic factors and alcohol use played an important role. Access to HCV therapy will be critical not only for preventing progression of liver disease but also potentially for improving metabolic factors. Efforts to expand treatment to those infected with HCV will need to incorporate interventions to reduce alcohol use in order to prevent further progression of liver disease.
